# An Arabidopsis Oxalyl-CoA Decarboxylase, AtOXC, Is Important for Oxalate Catabolism in Plants

**DOI:** 10.3390/ijms22063266

**Published:** 2021-03-23

**Authors:** Justin Foster, Ninghui Cheng, Vincent Paris, Lingfei Wang, Jin Wang, Xiaoqiang Wang, Paul A. Nakata

**Affiliations:** 1 USDA/ARS Children’s Nutrition Research Center, Department of Pediatrics, Baylor College of Medicine, Houston, TX 77030, USA; jf90@hotmail.com (J.F.); ncheng@bcm.edu (N.C.); 2 BioDiscovery Institute, University of North Texas, Denton, TX 76203, USA; vincentparis@my.unt.edu (V.P.); xiaoqiang.wang@unt.edu (X.W.); 3 Department of Pharmacology and Chemical Biology, Baylor College of Medicine, Houston, TX 77030, USA; lingfei.wang@bcm.edu (L.W.); wangj@bcm.edu (J.W.)

**Keywords:** oxalate, catabolism, decarboxylase

## Abstract

Considering the widespread occurrence of oxalate in nature and its broad impact on a host of organisms, it is surprising that so little is known about the turnover of this important acid. In plants, oxalate oxidase is the most well-studied enzyme capable of degrading oxalate, but not all plants possess this activity. Recently, acyl-activating enzyme 3 (AAE3), encoding an oxalyl-CoA synthetase, was identified in Arabidopsis. This enzyme has been proposed to catalyze the first step in an alternative pathway of oxalate degradation. Since this initial discovery, this enzyme and proposed pathway have been found to be important to other plants and yeast as well. In this study, we identify, in Arabidopsis, an oxalyl-CoA decarboxylase (AtOXC) that is capable of catalyzing the second step in this proposed pathway of oxalate catabolism. This enzyme breaks down oxalyl-CoA, the product of AtAAE3, into formyl-CoA and CO_2_. AtOXC:GFP localization suggested that this enzyme functions within the cytosol of the cell. An *Atoxc* knock-down mutant showed a reduction in the ability to degrade oxalate into CO_2_. This reduction in AtOXC activity resulted in an increase in the accumulation of oxalate and the enzyme substrate, oxalyl-CoA. Size exclusion studies suggest that the enzyme functions as a dimer. Computer modeling of the AtOXC enzyme structure identified amino acids of predicted importance in co-factor binding and catalysis. Overall, these results suggest that AtOXC catalyzes the second step in this alternative pathway of oxalate catabolism.

## 1. Introduction

Oxalate is the simplest of the dicarboxylic acids. Its biosynthesis in plants has been proposed to occur via multiple pathways. Isocitrate, glycollate, glyoxylate, oxaloacetate, and ascorbate have all been suggested as possible precursors to this organic acid [1]. Of these precursors, ascorbate has been considered the primary substrate for the biosynthesis of oxalate utilized in the formation of the calcium oxalate crystal [1].

In plants, oxalate has been shown to perform various functions including metal tolerance, ion balance, calcium regulation, and defense against insects [1,2,3]. Although the ability to produce ample amounts of oxalate can provide many beneficial functions to the plant, uncontrolled or prolonged exposure to this strong organic acid can cause multiple physiological problems. Such problems can result from a disruption of membrane integrity, disruption of mitochondrial metabolism, metal precipitation, and free radical formation [4].

The toxic attributes of oxalate are utilized by some phytopathogens for host infection. For example, the phytopathogen *Sclerotinia sclerotiorum* secretes oxalate, a known pathogenicity factor, to assist entry into the cells by stimulating stomatal opening, interfering with cell wall structure, inducing low-pH-activated pectolytic enzymes, and acting as an elicitor of programmed cell death [5,6,7,8,9,10,11,12,13]. As a result of the detrimental effects of this acid, plants must be able to control the amount of tissue oxalate in order to maintain proper cellular metabolism and overall plant health. It has been reported that some plants contain an enzyme, oxalate oxidase, which is capable of degrading oxalate into CO_2_ and H_2_O_2_ [14,15]. This activity has been shown to be important in stress responses such as defending plants against oxalate-secreting fungal phytopathogens (e.g., *S. sclerotiorum*) where the enzyme is targeted to the cell wall. Although oxalate oxidase activity has been detected in monocots [16,17], this activity appears to be absent in other plants [18,19], suggesting the possible existence of an alternative mechanism to degrade oxalate.

Such an alternative pathway of oxalate catabolism was proposed by Giovanelli and Tobin over 50 years ago [20]. Giovanelli and Tobin suggested a pathway of oxalate degradation in which oxalate was degraded to CO_2_ in a CoA- and ATP-dependent manner based on studies utilizing ^14^C-oxalate and partially purified extracts from pea (*Pisum sativum*). With no genes identified to support the existence of this pathway of oxalate catabolism, the pathway has remained overlooked for decades. Investigation into this pathway was revitalized upon the discovery that the Arabidopsis AAE3 gene encoded the elusive oxalyl-CoA synthetase, an enzyme capable of catalyzing the first step in this alternative pathway of oxalate catabolism [21]. AAE3 is a member of a large superfamily of acyl-activating enzymes (AAEs) in Arabidopsis [22,23]. The existence of such a catabolic pathway in Arabidopsis provided an explanation for its lack of oxalate oxidase activity. Since this initial discovery, it now appears that the CoA-dependent pathway of oxalate catabolism functions in other plants [24,25] and even microbes such as *Saccharomyces cerevisiae* [26]. Such findings lend credence to the importance of this pathway across species.

Although this CoA-dependent pathway of oxalate catabolism is important across species, the enzymes catalyzing the remaining steps in this pathway remain largely unknown and uncharacterized. Recently, an enzyme encoding oxalyl-CoA decarboxylase 1 was reported in maize [27]. Such an activity is capable of catalyzing the second step in the CoA pathway of oxalate catabolism. In support of this hypothesis, maize *ocd1* plants showed elevated oxalate concentrations in seeds. It is unknown whether a homolog of this enzyme capable of catalyzing the second step in the CoA pathway of oxalate catabolism is present in other plants. Direct assignment of this enzyme to the CoA-dependent pathway of oxalate catabolism, general features of this enzyme, and its importance to plant growth and development are also lacking.

In this study, we advance our understanding of this CoA-dependent pathway of oxalate catabolism by identifying and partially characterizing the gene and encoded enzyme activity responsible for catalyzing the second step in this pathway in Arabidopsis. Bioinformatic, biochemical, genetic, and molecular studies revealed that AtOXC encodes an oxalyl-CoA decarboxylase capable of catalyzing the conversion of oxalyl-CoA to formyl-CoA. Radiolabeled oxalate tracer studies suggest that the AtOXC catalyzes the second step in the CoA-dependent pathway of oxalate degradation. Oxalate assays support biological roles for AtOXC in the degradation of oxalate whether derived from exogenous or endogenous sources.

## 2. Results

### 2.1. Identification and Cloning of an Arabidopsis OXC Homolog

**Figure 1:**
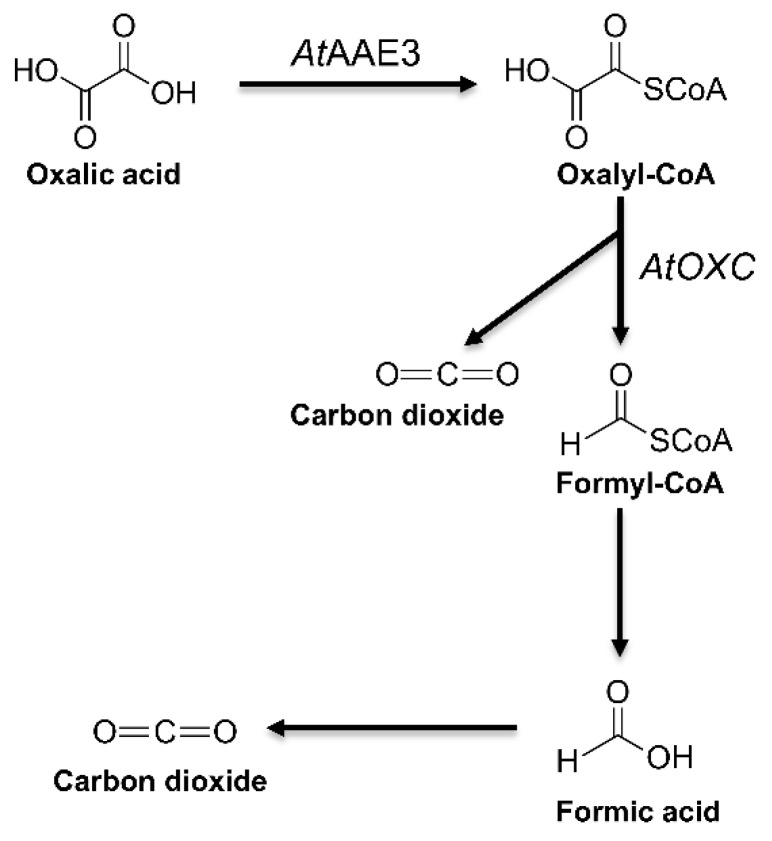
Proposed pathway of oxalate catabolism. AtAAE3, acyl-activating enzyme 3, which possesses an oxalyl-CoA synthetase activity [21], AtOXC, oxalyl-CoA decarboxylase (this study), and enzymes catalyzing the last two steps remain to be determined.

**Figure 2:**
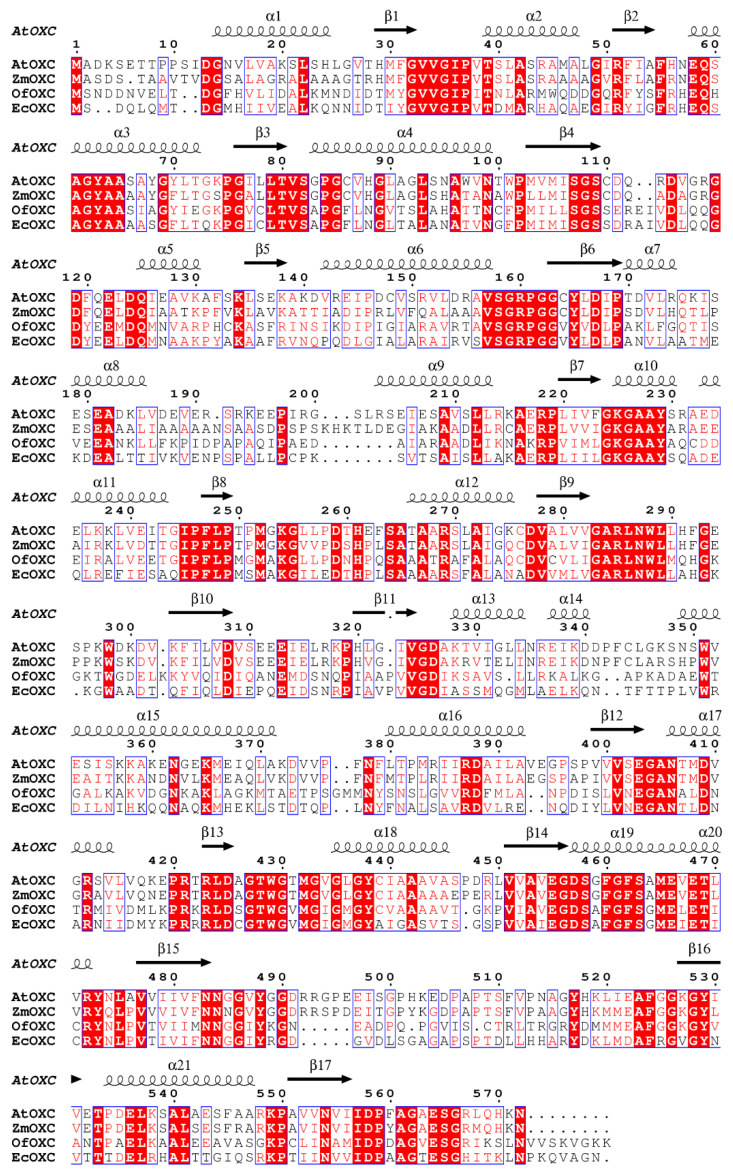
Comparison of the predicted amino acid sequences of OXC from plants and bacteria. Sequence alignment of OXCs from *Arabidopsis thaliana*, *Zea mays*, *Oxalobacter formigenes*, and *E. coli*. The secondary structure elements observed in the AtOXC modeled structure are shown above the alignment. Conserved residues are highlighted.

Our previous work proposed the existence of an oxalate catabolism pathway in dicot plants such as *Arabidopsis thaliana* (Figure 1) and demonstrated that an oxalyl-CoA synthetase (AAE3) acted as a key rate-limiting enzyme in this pathway [21]. Through co-expression analysis, using the NCBI protein blast program ATTEDII co-expression database (www.atted.bio.titech.ac.jp (accessed on 1 March 2012); [28]) and Gene Angler (http://bbc.botany.utoronot.ca (accessed on 1 March 2012); [29]) programs, an Arabidopsis *OXC* homolog (At5g17380) was found to be co-expressed with the *AtAAE3* gene and shared an overall sequence identity 40% and 43% (Figure 2), respectively, with the *Oxalobacter formigenes* OXC (OfOXC) [30], and the *Escherichia coli* OXC [31]. This putative AtOXC also shared 73% identity with oxalyl-CoA decarboxylase from corn (Figure 2) that was recently reported [27].

### 2.2. Arabidopsis OXC Possesses an Oxalyl-CoA Decarboxylase Activity

**Figure 3:**
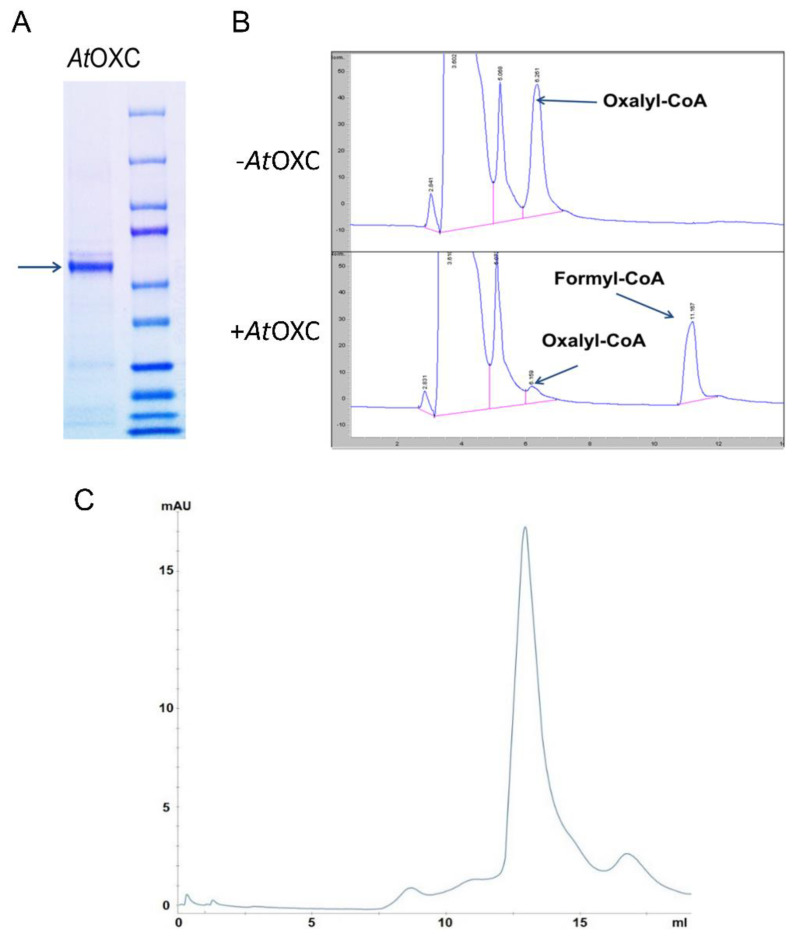
Biochemical analysis of AtOXC. (**A**) SDS-PAGE gel of nickel-affinity-purified His-AtOXC protein (**left**) and molecular weight markers (**right**). (**B**) HPLC analysis of enzyme reaction mix without and with added AtOXC. (**C**) Size exclusion chromatography of AtOXC using a Superdex 200 Increase 10/300 GL column. A dimeric form of AtOXC was detected during gel filtration using an ÄKTA purifier (GE Healthcare).

To determine whether this putative AtOXC could catalyze the decarboxylation of oxalyl-CoA to formyl-CoA, we first expressed a His-tagged AtOXC in *E. coli*. This His-tagged fusion protein was purified by nickel-affinity chromatography and was estimated to be about 90% pure via SDS-PAGE (Figure 3A).

To assess whether the AtOXC possessed enzymatic activity capable of catalyzing the second step in the CoA-dependent pathway, an aliquot of this purified preparation was tested to determine if it could produce formyl-CoA utilizing the products generated in the first step in the oxalate catabolism pathway as substrate. Thus, the oxalyl-CoA was first generated by mixing oxalate, CoA, and the purified AAE3 enzyme. After removal of the AAE3 enzyme by filtration, the second step in the proposed oxalate catabolism pathway then was initiated by the addition of the purified AtOXC and thiamine pyrophosphate. The production of formyl-CoA was monitored using HPLC. This analysis showed the appearance of the product (formyl-CoA) and disappearance of the substrate (oxalyl-CoA) in an AtOXC-dependent manner (Figure 3B), revealing that AtOXC does indeed encode an activity capable of catalyzing the second step in the proposed pathway of oxalate catabolism (Figure 1).

To determine the native form of the AtOXC enzyme, size exclusion analysis was performed by gel filtration through a Superdex 200 increase 10/300 GL column. The molecular weight of the monomeric form of AtOXC was determined to be around 61.5 kDa based on both SDS-PAGE fractionation and calculations based on the amino acid composition of the AtOXC enzyme. Elution of AtOXC from the gel filtration column was at 13.0 mL while the reference protein standards of aldolase (158 kDa) and conalbumin (75 kDa) eluted at 12.6 mL and 14.0 mL, respectively. This finding suggested that AtOXC functions as a dimer.

### 2.3. AtOXC Structural Model

**Figure 4:**
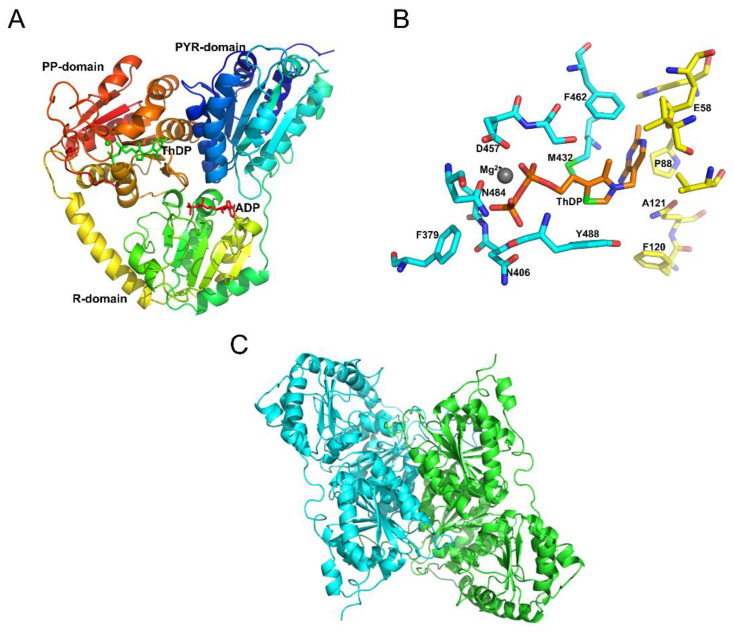
Molecular modeling of AtOXC. (**A**) A modeled structure of AtOXC docked with cofactors ThDP and Mg^2+^ ion, and activator ADP. Both ThDP (**green**) and ADP (**red**) are shown as stick models, and Mg^2+^ ion is shown as a sphere model in green. (**B**) Binding site of cofactors ThDP and Mg^2+^ ion. ThDP is shown as an orange stick model. Selected protein residues within the binding pocket are labeled and shown as stick models in cyan for residues from one subunit and yellow for residues from other subunit, and Mg^2+^ ion is shown as a gray sphere model. (**C**) Dimeric model of AtOXC. The two monomers are shown in green and cyan, respectively.

The AtOXC homology model was generated utilizing the known structures of the *E. coli* and *Oxalobacter formigenes* OXCs (PDB IDs: 2q29 and 2c31). Similar to the structures of *E. coli* and *O. formigenes* OXCs, the AtOXC model contains three domains. These domains consist of the α/β type fold, including the N-terminal PYR domain, the middle R domain, and the C-terminal PP domain (Figure 4A).

In the structure of the *OfOXC,* three residues, Tyr120, Glu121, and Tyr488, reside in the putative active site and are thought to be important for catalysis. The three corresponding residues in the AtOXC are Phe120, Gln121, and Tyr488, respectively, where Phe120 may contribute to a hydrophobic environment for the stabilization of the ThDP cofactor (Figure 4B). The model predicts that these amino acids come from two AtOXC subunits, suggesting that this enzyme functions as a dimer (Figure 4C).

Based on this model, we hypothesize that the ThDP cofactor resides between the PYR and PP domains of the two subunits, with its phosphate group interacting with Phe379 and Asn 406 and its pyrimidine ring interacting with Glu58 and His88 of the other subunit (Figure 4B). The divalent Mg^2+^ ion cofactor could also interact with Asp457 and Asn484. The activator, ADP, is predicted to sit in a cleft of the PYR, R, and PP domain of the same subunit where it can interact with neighboring hydrophilic residues such as Arg160, Lys225, Arg285, Asp308, and/or Thr428. Future mutagenesis studies will help to confirm the roles of these amino acids in ligand binding and catalysis.

### 2.4. AtOXC Gene Expression and Protein Subcellular Localization

**Figure 5:**
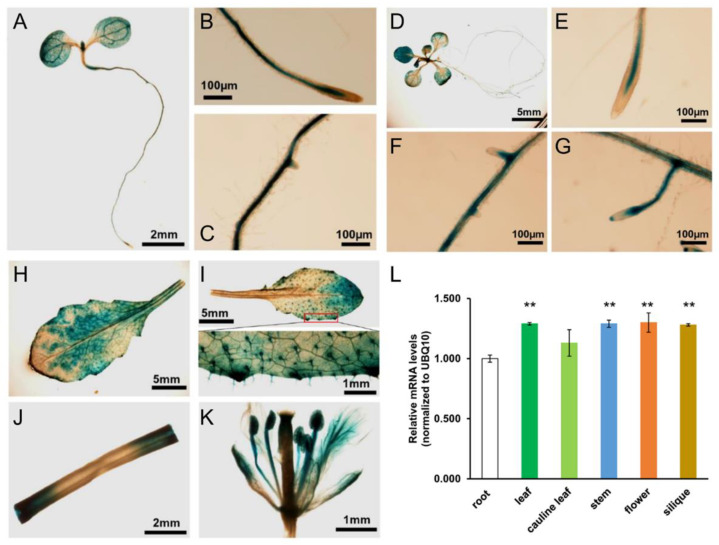
*AtOXC* expression in plants. *AtOXC*::Gus staining in 1-week-old seedlings (**A**), primary root (**B**), and lateral root (**C**). *AtOXC*::Gus staining in 2-week-old seedlings (**D**), primary root (**E**), and lateral roots (**F**,**G**). *AtOXC*::Gus staining in mature leaves (**H**,**I**), stem (**J**), and flower (**K**). (**L**) qRT-PCR analysis of *AtOXC* expression in different tissues of Arabidopsis. Ubiquitin 10 (*UBQ10)* was used as an internal control. Student’s *t-*test, *n* = 6, ** *p* < 0.01, indicating a significant difference between various tissues vs. roots.

GUS staining of *AtOXCp*::GUS transgenic plants and qPCR analysis of *AtOXC* expression revealed that *AtOXC* was ubiquitously expressed in Arabidopsis (Figure 5). In one-week-old seedlings, GUS staining was mainly observed in the vascular bundle of cotyledons and roots (Figure 5A–C). At two weeks of age, GUS expression was detected in the mesophyll of true leaves in addition to the vascular bundles (Figure 5D–G). In the mature leaf, strong GUS staining was visible in mesophyll and epidermal cells, including the trichromes (Figure 5H,I). In stems, strong GUS expression was concentrated at the cut edge of the tissue (Figure 5J). *AtOXCp*::GUS was highly expressed in flowers, especially in the petal and stamen (Figure 5K). Among the different tissues, roots showed the lowest expression of *AtOXC* (Figure 5L).

**Figure 6:**
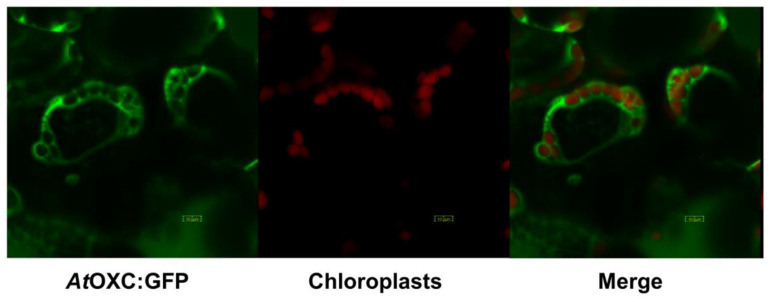
Subcellular localization of AtOXC-GFP. AtOXC-GFP expression in leaves of *A. thaliana* (**left**), chloroplast autofluorescence (**middle**), and merge of AtOXC-GFP and autofluorescence (**right**). Bar = 10 µm.

To investigate the subcellular localization of AtOXC, we expressed a GFP-AtOXC fusion protein in Arabidopsis and assessed its intracellular localization using confocal microscopy. As evidenced by its fluorescent pattern in comparison to the pattern exhibited by a free GFP control (data not shown), the GFP-AtOXC fusion protein was observed to reside within the cytoplasm of the cell (Figure 6).

### 2.5. Deletion of AtOXC Leads to Embryonic Lethality

As a step toward elucidating the physiological function of AtOXC in vivo, two transfer DNA (T-DNA) insertional mutant lines, Salk 142717 and SAIL 343D06, were screened for the *Atoxc* null allele. No *Atoxc* T-DNA insertion was found in the plants from the Salk 142717 seed pool. A T-DNA insertion in *Atoxc* was identified in the SAIL 343D06 line (*Atoxc-2*) (Supplementary Material Figure S1). The progeny from the identified heterozygous line, however, exhibited a segregation ratio of 1:1 (Basta resistant vs. Basta sensitive) in its progeny (Table 1), suggesting that *Atoxc-2* contained a defect in gametophyte development. To further determine which gametophyte was defective, reciprocal crosses between wild type (Col-0) and the heterozygous *atoxc-2* were performed. Progeny analysis revealed that about 94% of the *atoxc-2* allele was able to pass to the next generation through the female gametes, while only 1% of the *atoxc-2* allele was passed to the progeny through the male gametes (Table 1). This finding indicated that the defect was most likely associated with pollen viability or development.

### 2.6. Reduction of AtOXC Impairs the Catabolism of Oxalate

**Figure 7:**
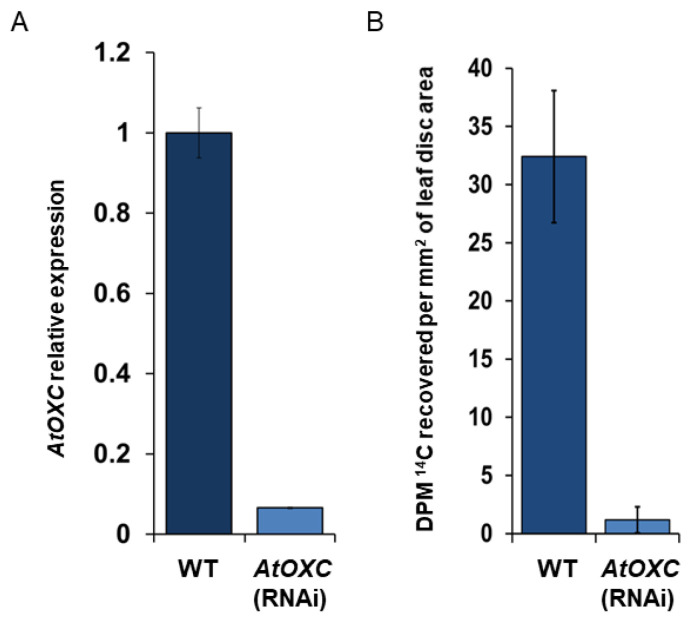
Measurement of oxalate degradation to CO_2_. (**A**) Relative *AtOXC* transcript levels in leaves of *AtOXC* knock-down mutant compared to WT as measured by qRT-PCR. (**B**) Radiolabeled CO_2_ measurements. WT and *AtOXC* knock-down leaf pieces were fed with 2.5 µCi of [^14^C]-oxalate along with 300 µM non-labeled oxalate. The ^14^CO_2_ evolved was captured using 1 M KOH and the relative radioactivity was measured.

To alleviate the observed *Atoxc* embryonic lethal phenotype, *AtOXC* RNAi knock-down mutants were generated by transforming WT Arabidopsis with a construct expressing inverted segments of the *AtOXC* coding region (stem) separated by a short segment of the Arabidopsis RTM intron (hairpin loop). Expression of this construct resulted in a reduction in *AtOXC* transcript abundance (Figure 7A) compared to WT. To determine if the AtOXC was required for oxalate catabolism in *Arabidopsis*, the *AtOXC* RNAi knock-down mutants and WT plants were utilized in a radiolabeled oxalate feeding experiment. Leaf discs cut from leaves of the *AtOXC ribonucleic acid interference (RNAi)* and WT plants were floated on an oxalate solution containing ^14^C-labeled oxalate in a sealed flask containing a CO_2_ trap. WT plants were found to be capable of degrading the ^14^C-oxalate, yielding ^14^CO_2_. The *AtOXC RNAi* mutants, however, had reduced ^14^CO_2_ emissions (Figure 7B). This reduction in ^14^CO_2_ emissions correlated with the reduction in *AtOXC* expression as measured by quantitative RT-qPCR (Figure 7A).

### 2.7. Reduction in AtOXC Results in the Accumulation of Oxalate and Oxalyl-CoA

**Figure 8:**
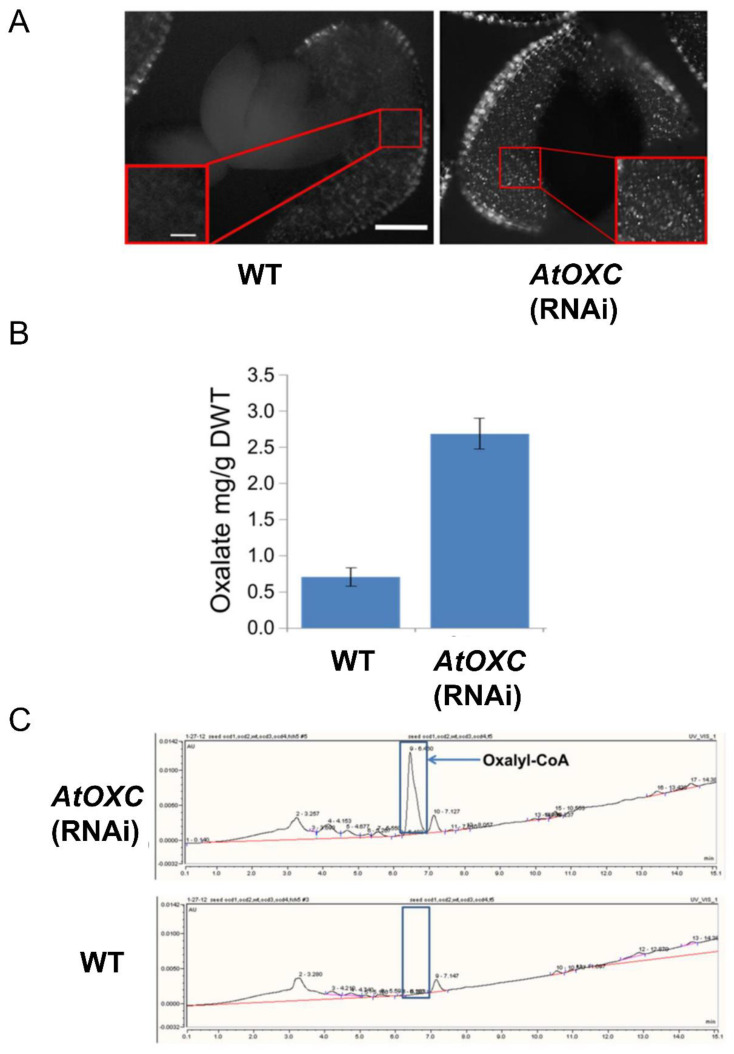
Assessment of oxalate and oxalyl-CoA accumulation in seeds. (**A**) Comparison of the calcium oxalate crystal phenotypes in seeds from WT and AtOXC knock-down plants. Crystals are bright spots denoted by arrows. Bar = 200 µm. (**B**) Total oxalate in seeds from WT and AtOXC knock-down plants. (**C**) Assessment of oxalyl-CoA accumulation in WT and AtOXC knock-down plants.

Recent studies have shown the importance of a functional AAE3 in maintaining low oxalate levels in the seeds of plants [21,24,32]. To investigate a role of AtOXC in regulating calcium oxalate accumulation in seeds of Arabidopsis, microscopic examinations were conducted. This analysis revealed the accumulation of crystals of calcium oxalate within the *AtOXC RNAi* seeds, while no crystals were observed in WT controls (Figure 8A). Oxalate measurements confirmed this observation, with higher oxalate concentrations measured in seeds from the *AtOXC* knock-down line compared to the corresponding control tissue (Figure 8B). HPLC analysis of the *AtOXC RNAi* and WT seeds showed an accumulation of an oxalyl-CoA peak which was absent in the WT controls (Figure 8C).

### 2.8. Reduction in AtOXC Results in an Increase in Sensitivity to Exogenous Oxalic Acid and Accumulation of Oxalyl-CoA

**Figure 9:**
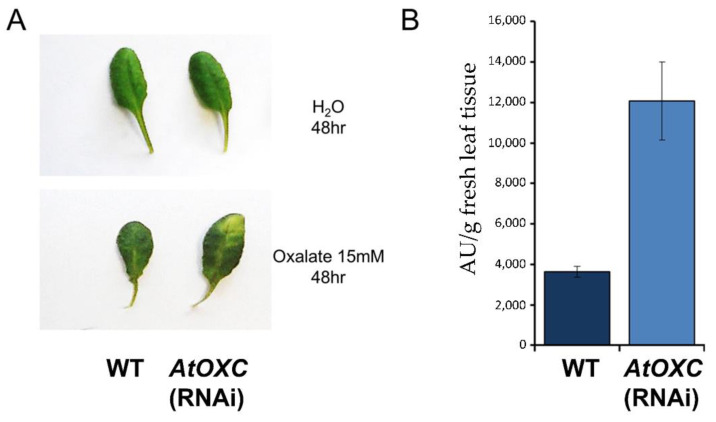
Response to exogenous application of oxalate. (**A**) WT and *AtOXC* knock-down mutant phenotypes in response to external oxalate exposure. (**B**) Accumulation of oxalyl-CoA accumulation in WT and *AtOXC* knock-down mutant leaves after external oxalate exposure.

Leaves of WT and *AtOXC* RNAi knock-down lines were exposed to exogenous oxalate. The *AtOXC* RNAi knock-down line was observed to display a higher sensitivity to the applied oxalate compared to WT, as indicated by the pronounced chlorosis (Figure 9A). A noticeable accumulation of oxalyl-CoA was also detected in the *AtOXC* RNAi plants compared to WT controls (Figure 9B).

## 3. Discussion

Although oxalic acid is common in nature and has a broad impact on plants, our understanding of the mechanisms regulating its turnover remains incomplete. Recently, a novel pathway of oxalate catabolism was suggested in Arabidopsis [21]. The existence of this catabolic pathway (Figure 1) is supported by the discovery of an oxalyl-CoA synthetase encoded by the *A. thaliana* AAE3 (AtAAE3) which has been shown to catalyze the first step in this pathway [21]. In this study, we have identified an Arabidopsis gene encoding an oxalyl-CoA decarboxylase (AtOXC) and demonstrated that AtOXC is capable of catalyzing the second step in the pathway of oxalate catabolism.

In contrast to AtAAE3, *AtOXC* is not part of a large gene family and appears to be conserved across species, including metazoans. Although the overall amino acid homology between plant AtOXC/ZmOXC and bacterial OfOXC/EcOXC is less than 50%, the predicted PP-, PYR-, ThDP-, ADP-, and R-domains are highly conserved (Figure 2 and Figure 3). In *O. formigenes*, OfOXC catalyzes the conversion of oxalyl-CoA into formyl-CoA and CO_2_ as part of an activation–decarboxylation pathway that allows *O. formigenes* to utilize oxalate as a sole carbon source [33]. Although the bacterial oxalate catabolism pathway [34] differs from the pathway (Figure 1) utilized by plants [21] and yeast [26], the OXC catalytic activity is conserved in plants such as Arabidopsis (Figure 4) and maize [27]. This conserved catalytic activity, however, appears to utilize different amino acid residues for cofactor and substrate binding, catalysis, and dimerization. For example, two out of three putative active site residues in the plant OXCs are different from the bacterial OXCs (Y120, Glu121, and Y488). Mutagenesis studies will help to confirm the roles of specific amino acids in ligand binding, catalysis, and dimerization.

Many plants accumulate oxalate, often in the form of the calcium oxalate crystal, where it has been shown to play beneficial roles in response to various adverse environmental conditions [1]. Not all plants, however, accumulate oxalate. Such plants include Arabidopsis and maize. Based on this finding, one might hypothesize that OXC expression would be prevalent throughout the plant, keeping tissue oxalate levels low in these non-oxalate-accumulating plants. *AtOXC* (Figure 5) and *ZmOXC* expression [27] was found in all tested tissues. *AtOXC* expression was abundant in cells of the vascular bundles and epidermis, including trichomes, which have been commonly observed to contain crystals of calcium oxalate in oxalate-accumulating plants [1]. High *AtOXC* expression was also observed in the apex of shoots and petals and the stamens of flowers (Figure 5). Interestingly, genetic analysis indicated that *AtOXC* loss of function plants were defective in male (pollen) gamete development, leading to embryonic lethality (Table 1). In oxalate-accumulating plants, oxalate crystals have been found in plant reproductive organs, including anthers [35,36]. Oxalate and calcium homeostasis in the male gametes are tightly controlled and this strict regulation may be critical for pollen growth, development, and pollination [36,37]. Whether AtOXC is directly involved in regulating pollen development is unclear and an area for future exploration.

*AtOXC* was found to be co-expressed with *AtAAE3* in Arabidopsis plants [21]. Sub-cellular localization of GFP-AtOXC in Arabidopsis showed that the enzyme functioned in the cytosol of cells (Figure 6). This finding is consistent with localization studies that place the AtAAE3 [21], as well as the AAE3 from other plants [24,25], within the cytosolic compartment. These findings suggest that AtAAE3 and AtOXC could act in a stepwise fashion catalyzing the first and the second reactions in the proposed CoA-dependent pathway of oxalate catabolism (Figure 1). In addition, AtOXC was found to be capable of generating formyl-CoA and CO_2_ directly from the reaction products of AtAAE3 (Figure 4).

Although the evidence shows that AtOXC is capable of catalyzing the second reaction in the proposed CoA-dependent pathway of oxalate catabolism, direct evidence assigning this activity to the proposed pathway of oxalate catabolism is lacking. This is an important step since there are many instances where an enzyme capable of catalyzing a particular reaction does not participate in a given pathway. As an example, there are a number of Ca ATPases that all possess the same enzyme activity (i.e., pump Ca) but each is a component of a different functional pathway. To determine if the AtOXC is required for oxalate catabolism in Arabidopsis, an *AtOXC* RNAi knock-down and WT plants were utilized in a radiolabeled oxalate feeding experiment (7A). WT plants were found to be capable of degrading the ^14^C-oxalate, yielding ^14^CO_2_. The *Atoxc* mutants, however, had reduced ^14^CO_2_ emissions (Figure 7B). This reduction in ^14^CO_2_ emissions correlated with the reduction in *AtOXC* gene expression as measured by quantitative RT-qPCR (Figure 7A). These results are consistent with the findings derived from a similar experiment comparing CO_2_ emissions from WT and the *Ataae3* mutant. Overall, these findings support a role for AtOXC in catalyzing the second step in a pathway of oxalate degradation to CO_2_ in *Arabidopsis.*

In further support, the reduction in *AtOXC* expression was found to increase the sensitivity of the knock-down plant to exogenous oxalic acid. Excess oxalate is known to be toxic to plants and enhanced oxalate degradation makes plants less sensitive to its toxicity [38,39]. A previous study [21] showed, utilizing the *Ataae3* plants, that a reduction in the ability to catabolize oxalate resulted in an increase in sensitivity to an exogenous source of oxalate, whether from a direct application or from an oxalate-secreting phytopathogen. Our results indicated that *AtOXC* RNAi plants responded in a manner similar to the response exhibited by *Ataae3* plants when challenged with an exogenous supply of oxalate (Figure 9).

Finally, studies have shown the importance of a functional AAE3 in maintaining low oxalate levels in the seeds of each of these plants [21,24]. Similarly, a role for AtOXC in regulating calcium oxalate accumulation in seeds of Arabidopsis was determined by microscopic examination. This analysis revealed the accumulation of crystals of calcium oxalate within the *Atoxc* seeds, while no crystals were observed in WT controls (Figure 8A). Oxalate measurements confirmed this observation, with higher oxalate concentrations measured in seeds from the *Atoxc* knock-down line compared to the corresponding control (Figure 8B). HPLC analysis of the *Atoxc* and WT seeds showed an accumulation of an oxalyl-CoA peak which was absent in the WT control (Figure 8C). Overall, these findings support a role for AtOXC in catalyzing the second step in a CoA-dependent pathway of oxalate catabolism in Arabidopsis.

## 4. Materials and Methods

### 4.1. AtOXC cDNA Isolation

Total RNA was extracted from leaves of Arabidopsis using TRIzol reagent (Life Technologies, Carlsbad, CA, USA) according to the manufacturer’s instructions. Total RNA was used for first-stand cDNA synthesis using oligo (dT) and Superscript III first strand synthesis supermix (Life Technologies, Carlsbad, CA, USA). The *AtOXC* coding sequence was amplified by PCR using a 4 μL aliquot of the reverse transcription reaction, gene specific primers, 5′-ATGGCGGATAAATCAGAAACC-3′ and 5′-TTAGTTCTTGTGCTGTAATCTCC-3′, and Platinum Taq DNA Polymerase High Fidelity (Life Technologies) according to the manufacturer’s instructions. All hybridization steps were performed using a PTC-2 thermal cycler (MJ Research, BioRad, Hercules, CA, USA). The PCR product was cloned using the pGEM-T Easy kit (Promega, Madison, WI, USA) according to the manufacturer’s instructions and verified by DNA sequencing (Eurofins Genomics, Louisville, KY, USA).

### 4.2. OXC Alignment and Homology Modeling

Multiple sequence alignment of OXCs from *Arabidopsis thaliana*, *Zea mays*, *Oxalobacter formigenes*, and *E. coli* was performed using ClustalX [40] and rendered by ESPript [41]. The comparative modeling of the AtOXC to the *E. coli* and *Oxalobacter formigenes* oxalyl CoA decarboxylase (PDB ID: 2q29, 2c31) structures was done utilizing the program MODELLER [42]. The three-dimensional structural model of AtOXC was generated based on optimal sequence alignment with CLUSTALX and the three-dimensional structure of templates. The ligand structure models were obtained by the superposition of the AtOXC model onto the *E. coli* oxalyl CoA decarboxylase containing co-factor (i.e., ThDP and Mg^2+^ and activator ADP) structure. The dimeric structural model of AtOXC was assembled with two monomeric structural models using the *Oxalobacter formigenes* oxalyl CoA decarboxylase dimeric structure (PDB ID: 2c31) as a reference. All structure models were analyzed using the graphics program COOT [43]. Figures were prepared using PyMOL (PyMOL Molecular Graphics System, Version 1.3, Schrödinger, LLC; https://pymol.org (accessed on 1 March 2012)).

### 4.3. His-Tagged AtOXC Recombinant Protein Purification

To create a His-tagged AtOXC fusion protein, the full-length AtOXC cDNA was amplified by PCR using the primers 5-CATATGCACCACCACCACCACCACAGCCAGGCGGATAAATCAGAAACCACT-3, which introduced an *Nde*I site and six histidine residues on its N-terminus, and 5-GAGCTCTTAGTTCTTGTGCTGTAATCTC-3, which contained an end terminal *Sac*I site. The PCR product was ligated into the plasmid vector pGEM-T Easy (Promega, Madison, WI, USA) and sequenced. The *Nde*I/*Sac*I His-AtOXC fragment was transferred from the pGEM-T Easy vector into the protein expression vector Pet-29a (Novagen, EMD Biosciences, Madison, WI, USA) using the same restriction sites. *Escherichia coli* strain BLR (DE3) competent cells (Novagen) were transformed with the N-terminal His-tagged AtOXC expression vector. A small culture of BLR (DE3) cells containing the AtOXC expression construct was grown overnight at 37 °C and used to inoculate 500 mL of Luria–Bertani medium. The large culture was incubated at 37 °C until it reached an OD_600nm_ of 0.4. To induce expression, IPTG was added to 1 mM, and the culture was grown for an additional 4 h at 30 °C. The cells were then collected by centrifugation and the cell pellet frozen. Affinity purification of the His-tagged AtOXC was performed as described in the Qiagen protein purification kit manual (Valencia, CA, USA). In brief, the bacterial cell pellet was thawed for 15 min on ice. The thawed cells then were resuspended in lysis buffer (50 mM NaH_2_PO_4_, 300 mM NaCl, and 10 mM imidazole, pH 8.0) supplemented with lysozyme (1 mg/mL) and benzonase, and incubated on ice for an additional 30 min, followed by sonication to lyse the cells. The extract then was cleared by centrifugation at 10,000× *g* for 25 min at 4 °C. The supernatant was collected and loaded onto a column packed with nickel–nitriloacetic acid agarose to bind the His-tagged AtOXC. The column was washed with wash buffer (50 mM NaH_2_PO_4_, 300 mM NaCl, and 20 mM imidazole at pH 8) and eluted using 1 mL of elution buffer (50 mM NaH_2_PO_4_, 300 mM NaCl, and 250 mM imidazole, pH 8.0). Salts were removed by passing the protein sample through a column packed with Sephadex G-25 (Sigma-Aldrich, St. Louis, MO, USA) and equilibrated with 100 mM Tris-HCl, pH 7.5. The protein concentration of the eluate was determined by a Bradford assay. An estimation of the molecular weight and purity of the affinity purified AtOXC sample was assessed by SDS-polyacrylamide gel and Coomassie Brilliant Blue R 250 staining.

### 4.4. Size Exclusion Analysis of AtOXC

Purified AtOXC and molecular weight size standards (75 kDa, conalbumin and 158 kDa, aldolase) were chromatographed on a Superdex 200 Increase 10/300 GL column equilibrated with 100 mM sodium phosphate buffer (pH 7.5), 100 mM NaCl, and 10 mM β-mercaptoethanol using an ÄKTA purifier (GE Healthcare, Chicago, IL, USA).

### 4.5. Assessment of AtOXC Activity

AtOXC enzyme activity was detected by HPLC. The enzyme reaction substrate, oxalyl CoA, was generated by the addition of 5 μg of purified AtAAE3 protein [21] to the buffered reaction mixture containing 0.1 M Tris-HCl (pH 8) or 0.1 M NaPO_4_ (pH 8), 2 mM dithiothreitol, 5 mM ATP, 10 mM MgCl_2_, and 0.5 mM CoA in a final volume of 800 μL. Once the reaction went to completion, the reaction mixture was filtered using an AmiconUltra 30 K centrifugal column (Merck Millipore Ltd., Billerica, MA, USA) to remove the AtAAE3 enzyme. Five micrograms of purified AtOXC protein were added to the filtered reaction mixture, along with thiamine pyrophosphate, to a concentration of 60 μM. Aliquots of this reaction before and after the addition of OXC were analyzed by HPLC to visualize the generation of formyl-CoA and depletion of oxalyl-CoA. The reaction was stopped by separating the enzyme from the assay mixture with the use of an Amicon Ultra 30 K centrifugal device (Merck Millipore Ltd., Billerica, MA, USA). Reaction products were analyzed by HPLC, as previously described [44]. In brief, CoA compounds were resolved using an Agilent 1100 HPLC (Agilent Technologies, Santa Clara, CA, USA) coupled to a photodiode array detector (Agilent 1100) at 254 nm with a C-18 reversed-phase Synergi 4μHydro-RP 80 A, 250 × 4.6 mm column (Phenomenex, Torrance, CA, USA) equilibrated with 86% buffer A (25 mM NaOAc, pH 4.5) and 14% buffer B (20 mM, NaOAc, pH 4.5, 20% CH_3_CN) at 0.75 mL/min. Following the injection of 15 μL of the reaction mixture, a 15 min linear gradient to 40% buffer B was initiated, followed by a step to 100% buffer B for two minutes, then a step back to 14% buffer B for the remaining time of the 28 min run.

### 4.6. Generation of AtOXCp::Gus Lines and Histochemical Analysis

A 917 bp DNA sequence upstream of the ATG of the *AtOXC* open reading frame (ORF) was amplified from genomic DNA using the *AtOXC* promoter forward and reverse primers (forward primer, 5-AAC TGC AGG TTT TTG TTTACA AAA AGA ACT CA-3; reverse primer, 5-CGTCTA GAG TTC TCT TGG ATT TGC TCA AAG-3). The PCR fragment was cloned into p3300 to replace the 35 S promoter, resulting in the plasmid pAtOXCp::Gus. Agrobacterium-mediated transformation of Arabidopsis plants was performed using the floral-dip method [45]. Histochemical analysis was performed following the published protocol [46].

### 4.7. Plant Growth Conditions, RNA Isolation, cDNA Synthesis, and qRT-PCR Analysis

Wild type (ecotype Columbia, Col-0) seeds were sown on commercial soil (Pro-Line, growing mix, C/20, Jolly Gardener, Oldcastle Lawn & Garden, Atlanta, GA, USA) in 8 cm square pots and grown in a walk-in growth chamber under long-day growing conditions (16/8 h photoperiod) at 22/18 °C. Mature tissues (rosette leaf, cauline leaf, stem, flower, silique, and root) from five-week-old flowering plants were collected and harvested for RNA isolation. Three independent samples (plants) were conducted for each tissue type. Total RNA was extracted from tissue samples using the QIAGEN RNeasy Plant Mini Kit. Five micrograms of total RNA were treated with DNase I and then 2 µg DNase I-treated RNA samples underwent reverse transcription to yield cDNA using random hexamers. The resulting cDNA was diluted to 250 ng/µL and 1 µL of cDNA was used as a template for each qPCR reaction. qRT-PCR was performed using the SYBR Green-based system on the Bio-Rad CFX96™ (Bio-Rad, Hercules, CA, USA). CFX Maestro Software by Bio-Rad was used for data collection and the expression change was calculated using the 2^−ΔΔCt^ method. Relative mRNA levels were normalized to UBQ10 expression as an internal reference. Primers used were *AtOXC* forward: 5-TCGCTGTTGAAGGAGACTCTG-3 and reverse: 5-CACAGCAAGATTGTATCGAACC-3 and *UBQ10* forward: 5-GCTTCGTTTTTATTATCTGTGCTTCTT-3 and reverse: 5-TCGCAGAACTGCACTAAACAGAGT-3.

### 4.8. Subcellular Localization of AtOXC

The *AtOXC* cDNA was amplified by PCR using the primers 5′-CACCATGGCGGATAAATCAGAAACCACTCCACCG-3′ and 5′-TTAGTTCTTGTGCTGTAATCTCCCACTCTCAGCACCAG-3′. The *AtOXC* cDNA was then ligated into the pENTR Directional Topo vector according to the manufacturer’s instructions (Life Technologies, Carlsbad, CA, USA). The cloned *AtOXC* cDNA was then recombined into the pB7WGF2 vector [47] using the Gateway LR clonase II enzyme mix (Cat No. 11791-00) according to the manufacturer’s instructions (Life Technologies) to generate a GFP-AtOXC expression construct. This expression construct was introduced into *A. tumefaciens* strain GV3101 and utilized to transform Arabidopsis as described above. Protein localization was investigated using an FV300 laser scanning confocal microscope (Olympus America Inc., New York, NY, USA) using an argon laser. A 488 nm excitation and a 505 to 530 nm emission filter set were utilized for GFP observation.

### 4.9. AtOXC T-DNA Insertional Mutant and Generation of Atoxc Knock-Down Mutant

To isolate *atoxc* alleles, two T-DNA insertional mutant lines were obtained from the Arabidopsis Biological Resource Center (Salk_142717, termed *atoxc-1* and Sail_343_D06, termed *atoxc-2*) [48] (Figure S1). A T-DNA left border primer (LBb1: 5-GCGTGGACCGCTTGCTGCA-3) and a reverse primer (OXCg-3: 5-ACC AGA GTC TCC TTC AAC AGC GAC AAC-3) were used for screening *atoxc-1* alleles. A forward primer (OXCgg-5: 5-GTT TGG TAA AGG AGC TGC GTA TTC GAG-3) and a reverse primer (OXCg-3) were used for wild type alleles. A T-DNA left border primer (LB3: 5-TAGCATCTGAATTTCATAACCAATCTCGATACAC-3) and a reverse primer (AtOXC-3: 5′-CGG CTC GAG TTA GTT CTT GTG CTG TAA TCT-3) were used for screening *atoxc-2* alleles. A forward primer (OXCg-5: 5-GGT ATC GGA AGG AGC TAA TAC AAT GGA TG-3) and AtOXC-3 were used for wild type alleles.

To create the RNAi knock-down construct, a hairpin loop, containing two complementary *AtOXC* sequences separated by Restricted Tobacco etch virus Movement (RTM) intron, was constructed. The Arabidopsis RTM was cloned into *Not*I and *Xba*I sites to make the pIntron vector, as previously described [24]. A 446 bp segment of the *AtOXC* gene was amplified using the *AtOXC* cDNA as template and the primers 5-TCCCCGCGGGTTTCGTTGCTGAGGAAAGC-3 and 5-ATAAGAATGCGGCCGCTCTTCGAAATCGATTCCACC-3, which introduce a *Sac*II and *Not*I site on the 5′ and 3′ end of the amplified fragment, respectively. The reverse complement of this 446 bp segment was generated using the same template cDNA and the primers 5-GCTCTAGATCTTCGAAATCGATTCCACC-3 and 5-GACTAGTGTTTCGTTGCTGAGGAAAGC-3, which introduced an *Xba*I and *Spe*I site on the 5′ and 3′ end of the fragment, respectively. The *Sac*I-*Not*I *AtOXC* fragment was cloned into the pIntron vector after digesting with same restriction sites. After amplification of this construct in DH5α, the *Spe*I-*Xba*I *AtOXC* fragment was cloned into the corresponding sites. In this construct, the two complementary *AtOXC* sequences were separated by 143 bp of the Restricted Tobacco etch virus Movement (RTM) intron to create a hairpin loop [49]. The *AtOXC*-intron-*AtOXC* sequence was liberated by digestion with *Bam*I/*Sac*I and used to replace the GUS gene in the pBI121 [50] expression construct. The resulting RNAi expression construct was transformed into *A*. *tumefaciens* strain GV3101 by electroporation. GV3101 cells containing the *AtOXC* RNAi construct were selected on kanamycin and utilized in the transformation of WT *Arabidopsis thaliana* using the floral-dip method [45]. The transgenic Arabidopsis plants were selected on Murashige and Skoog basal medium (MS) plates containing 50 mg/L kanamycin, as previously described [45].

### 4.10. T-DNA Transmission Analysis

For genetic transmission analysis, heterozygous *atoxc*-*2* was reciprocally crossed with the wild type (Col-0 ecotype). The backcrossed F1 seeds were germinated and grown on one-half strength MS plus 0.5% sucrose medium supplemented with 15 µg/mL Basta (Glufosinate–ammonium). After 10 days, the numbers of Basta-resistant and Basta-sensitive seedlings were counted for transmission efficiency. In addition, F2 seeds from self-pollination of heterozygous *atoxc-2* plants were germinated and grown in soil for two weeks, and then sprayed with Basta solution. The number of Basta-resistant and Basta-sensitive seedlings were calculated.

### 4.11. Radiolabeled Oxalate Feeding

Leaf discs of *A. thaliana* wild type and *AtOXC* RNAi knock-down lines were isolated using an 8.5 mm borer. The leaf discs were then placed in an Erlenmeyer flask containing 5 mL of MS media, pH 5.7 [51] supplemented with 0.5% sucrose, 0.05% 2-(N-morpholino)ethanesulfonic acid (MES), 500 μM oxalate, and 5 μCi of [^14^C]-oxalate (American Radiolabeled Chemicals, St. Louis, MO, USA). A glass vial containing 500 μL of 1M KOH was utilized as a CO_2_ trap and the flask sealed with a neoprene stopper. The flasks were slowly shaken at room temperature for 5 h and the reaction stopped by the addition of 1 mL of 0.25 M HCl that was injected through the stopper. The leaf discs were shaken for an additional 10 min and the radiolabeled CO_2_ trapped in the KOH measured using a Tricarb 2500TR liquid scintillation analyzer (Packard Bioscience Co., Meriden, CT, USA).

### 4.12. Oxalate Sensitivity Assay

Leaves were excised from wild type and *Atoxc* mutant plants. Petioles were cut underwater using a razor blade. The petioles of the leaves then were placed in 500 μL of distilled water or 15 mM oxalate in 15 mL Falcon tubes and the tubes sealed with micropore tape and placed under 115 μmol photons/m^2^/s light for 48 h. Photographs were taken to document the leaf phenotypes and oxalate measurements were conducted as described below.

### 4.13. Microscopic Analysis of Calcium Oxalate Crystal Phenotype

Seeds were cleared in 95% (*v*/*v*) ethanol, equilibrated with water, and then visually inspected for calcium oxalate crystal deposition using light microscopy and crossed polarizers. Images of whole-tissue mounts were captured using a CCD72 camera mounted on a Zeiss Axiophot light microscope (ZEISS Microscopy, Jena, Germany).

### 4.14. Measurement of Seed Oxalate Concentrations

Oxalate concentrations were measured by HPLC. Mature seeds were harvested from three independently grown sets of plants and ground using a mortar and pestle. Oxalate extraction was performed as described previously [3] and the samples filtered (0.2 μm) and analyzed for oxalate by HPLC (Agilent 1100) coupled to a photodiode array detector (Agilent 1100) with a Bio-Rad Aminex HPX-87H ion exclusion column (Hercules, CA, USA) (300 × 7.8 mm, 0.6 mL min^−1^, 35 °C). External standards of oxalate were used to determine sample oxalate concentrations.

## 5. Conclusions

In this study, we identify a gene encoding an oxalyl-CoA decarboxylase activity that is capable of catalyzing the second step in a novel CoA-dependent pathway of oxalate catabolism. Enzyme assays showed AtOXC encodes an oxalyl-CoA decarboyxalase that is capable of catalyzing the conversion of oxalyl-CoA to formyl-CoA and CO_2_. Radiotracer studies allowed the placement of AtOXC in the CoA-dependent pathway of oxalate catabolism. AtOXC was found to be essential in the degradation of oxalate, whether from an endogenous or exogenous source. The ability to degrade endogenous oxalate was found to be important in the regulation of druse crystal accumulation and in the production of viable pollen, while the ability to degrade exogenous oxalate has been shown to be important in defense against oxalate-secreting phytopathogens [21,24]. Because of these two functional roles, further study of this CoA-dependent pathway of oxalate degradation could lead to the development of new strategies to improve the nutrition quality and production of crops.

## Figures and Tables

**Table 1:** T-DNA transmission analysis of *atoxc-2*.

Crossing	Resistant	Sensitive	Ratio	Transmission Efficiency (%)
*atoxc-2* self-fertilized	228 (Bar^R^)	233 (Bar^S^)	0.979	/
*atoxc-2* (♀) × wild type (♂)	100 (Bar^R^)	107 (Bar^S^)	0.935	93.5
wild type (♀) × *atoxc-2* (♂)	1 (Bar^R^)	76 (Bar^S^)	0.013	1.3

## Data Availability

Data is contained within the article or Supplementary Material.

## Supplementary Materials

Supplementary material can be found at https://www.mdpi.com/1422-0067/22/6/3266/s1.

## References

[1] Franceschi V.R., Nakata P.A. (2005). Calcium Oxalate in Plants: Formation and Function. Annu. Rev. Plant Biol..

[2] Nakata P.A. (2003). Advances in our understanding of calcium oxalate crystal formation and function in plants. Plant Sci..

[3] Nakata P.A. (2012). Engineering Calcium Oxalate Crystal Formation in Arabidopsis. Plant Cell Physiol..

[4] Scheid C., Koul H., Hill W.A., Luber-Narod J., Kennington L., Honeyman T., Jonassen J., Menon M. (1996). Oxalate toxicity in LLC-PK1 cells: Role of free radicals. Kidney Int..

[5] Bateman D.F., Beer S.V. (1965). Simultaneous Production and Synergistic Action of Oxalic Acid and Polygalacturonase during Pathogenesis by Sclerotium Rolfsii. Phytopathology.

[6] Dong X., Ji R., Guo X., Foster S.J., Chen H., Dong C., Liu Y., Hu Q., Liu S. (2008). Expressing a gene encoding wheat oxalate oxidase enhances resistance to Sclerotinia sclerotiorum in oilseed rape (*Brassica napus*). Planta.

[7] Guimarães R.L., Stotz H.U. (2004). Oxalate Production by Sclerotinia sclerotiorum Deregulates Guard Cells during Infection. Plant Physiol..

[8] Lumsden R.D. (1976). Pectolytic enzymes of Sclerotinia sclerotiorum and their localization in infected bean. Can. J. Bot..

[9] Hegedus D.D., Rimmer S.R. (2005). Sclerotinia sclerotiorum: When “to be or not to be” a pathogen?. FEMS Microbiol. Lett..

[10] Kim K.S., Min J.-Y., Dickman M.B. (2008). Oxalic Acid Is an Elicitor of Plant Programmed Cell Death during Sclerotinia sclerotiorum Disease Development. Mol. Plant-Microbe Interact..

[11] Dutton M.V., Evans C.S. (1996). Oxalate production by fungi: Its role in pathogenicity and ecology in the soil environment. Can. J. Microbiol..

[12] Williams B., Kabbage M., Kim H.-J., Britt R., Dickman M.B. (2011). Tipping the Balance: Sclerotinia sclerotiorum Secreted Oxalic Acid Suppresses Host Defenses by Manipulating the Host Redox Environment. PLoS Pathog..

[13] Franceschi V.R., Loewus F.A., Khan S.R. (1995). Oxalate biosynthesis and function in plants and fungi. Calcium Oxalate in Biological Systems.

[14] Lane B.G. (2002). Oxalate, Germins, and Higher-Plant Pathogens. IUBMB Life.

[15] Svedružić D., Jonsson S., Toyota C.G., Reinhardt L.A., Ricagno S., Lindqvist Y., Richards N.G. (2005). The enzymes of oxalate metabolism: Unexpected structures and mechanisms. Arch. Biochem. Biophys..

[16] Lane B., Dunwell J., Ray J., Schmitt M., Cuming A. (1993). Germin, a protein marker of early plant development, is an oxalate oxidase. J. Biol. Chem..

[17] Druka A., Kudrna D., Kannangara C.G., Von Wettstein D., Kleinhofs A. (2002). Physical and genetic mapping of barley (*Hordeum vulgare*) germin-like cDNAs. Proc. Natl. Acad. Sci. USA.

[18] Membré N., Berna A., Neutelings G., David A., David H., Staiger R., Vásquez J.S., Raynal M., Delseny M., Bernier F. (1997). cDNA sequence, genomic organization and differential expression of three Arabidopsis genes for germin/oxalate oxidase-like proteins. Plant Mol. Biol..

[19] Membré N., Bernier F., Staiger D., Berna A. (2000). Arabidopsis thaliana germin-like proteins: Common and specific features point to a variety of functions. Planta.

[20] Giovanelli J., Tobin N.F. (1961). Adenosine Triphosphate- and Coenzyme A-dependent Decarboxylation of Oxalate by Extracts of Peas. Nat. Cell Biol..

[21] Foster J., Kim H.U., Nakata P.A., Browse J. (2012). A Previously Unknown Oxalyl-CoA Synthetase Is Important for Oxalate Catabolism in Arabidopsis. Plant Cell.

[22] Shockey J.M., Fulda M.S., Browse J. (2003). Arabidopsis Contains a Large Superfamily of Acyl-Activating Enzymes. Phylogenetic and Biochemical Analysis Reveals a New Class of Acyl-Coenzyme A Synthetases. Plant Physiol..

[23] Shockey J., Browse J. (2011). Genome-level and biochemical diversity of the acyl-activating enzyme superfamily in plants. Plant J..

[24] Foster J., Luo B., Nakata P.A. (2016). An Oxalyl-CoA Dependent Pathway of Oxalate Catabolism Plays a Role in Regulating Calcium Oxalate Crystal Accumulation and Defending against Oxalate-Secreting Phytopathogens in Medicago truncatula. PLoS ONE.

[25] Lou H.Q., Fan W., Xu J.M., Gong Y.L., Jin J.F., Chen W.W., Liu L.Y., Hai M.R., Yang J.L., Zheng S.J. (2016). An Oxalyl-CoA Synthetase Is Involved in Oxalate Degradation and Aluminum Tolerance. Plant Physiol..

[26] Foster J., Nakata P.A. (2014). An oxalyl-CoA synthetase is important for oxalate metabolism inSaccharomyces cerevisiae. FEBS Lett..

[27] Yang J., Fu M., Ji C., Huang Y., Wu Y. (2018). Maize Oxalyl-CoA Decarboxylase1 Degrades Oxalate and Affects the Seed Metabolome and Nutritional Quality. Plant Cell.

[28] Obayashi T., Hayashi S., Saeki M., Ohta H., Kinoshita K. (2008). ATTED-II provides coexpressed gene networks for Arabidopsis. Nucleic Acids Res..

[29] Toufighi K., Brady S.M., Austin R., Ly E., Provart N.J. (2005). The Botany Array Resource: E-Northerns, Expression Angling, and promoter analyses. Plant J..

[30] Lung H.Y., Baetz A.L., Peck A.B. (1994). Molecular cloning, DNA sequence, and gene expression of the oxalyl-coenzyme A decarboxylase gene, oxc, from the bacterium Oxalobacter formigenes. J. Bacteriol..

[31] Makkapati S., D’Agati V.D., Balsam L. (2018). “Green Smoothie Cleanse” Causing Acute Oxalate Nephropathy. Am. J. Kidney Dis..

[32] Cheng N., Foster J., Mysore K.S., Wen J., Rao X., Nakata P.A. (2018). Effect of Acyl Activating Enzyme (AAE) 3 on the growth and development of Medicago truncatula. Biochem. Biophys. Res. Commun..

[33] Baetz A.L., Allison M.J. (1989). Purification and characterization of oxalyl-coenzyme A decarboxylase from Oxalobacter formigenes. J. Bacteriol..

[34] Stewart C.S., Duncan S.H., Cave D.R. (2004). Oxalobacter formigenes and its role in oxalate metabolism in the human gut. FEMS Microbiol. Lett..

[35] Coté G.G., Gibernau M. (2012). Distribution of calcium oxalate crystals in floral organs of Araceae in relation to pollination strategy. Am. J. Bot..

[36] Gębura J., Winiarczyk K. (2016). A study on calcium oxalate crystals in Tinantia anomala (Commelinaceae) with special reference to ultrastructural changes during anther development. J. Plant Res..

[37] Paiva E.A.S. (2019). Are calcium oxalate crystals a dynamic calcium store in plants?. New Phytol..

[38] Chakraborty N., Ghosh R., Ghosh S., Narula K., Tayal R., Datta A., Chakraborty S. (2013). Reduction of Oxalate Levels in Tomato Fruit and Consequent Metabolic Remodeling Following Overexpression of a Fungal Oxalate Decarboxylase. Plant Physiol..

[39] Kumar V., Chattopadhyay A., Ghosh S., Irfan M., Chakraborty N., Chakraborty S., Datta A. (2016). Improving nutritional quality and fungal tolerance in soya bean and grass pea by expressing an oxalate decarboxylase. Plant Biotechnol. J..

[40] Chenna R. (2003). Multiple sequence alignment with the Clustal series of programs. Nucleic Acids Res..

[41] Gouet P., Courcelle E. (2002). ENDscript: A workflow to display sequence and structure information. Bioinformatics.

[42] Fiser A., Šali A. (2003). Modeller: Generation and Refinement of Homology-Based Protein Structure Models. Methods Enzymol..

[43] Emsley P., Cowtan K. (2004). Coot: Model-building tools for molecular graphics. Acta Crystallogr. Sect. D Biol. Crystallogr..

[44] Demoz A., Garras A., Asiedu D.K., Netteland B., Berge R.K. (1995). Rapid method for the separation and detection of tissue short-chain coenzyme A esters by reversed-phase high-performance liquid chromatography. J. Chromatogr. B Biomed. Sci. Appl..

[45] Clough S.J., Bent A.F. (1998). Floral dip: A simplified method forAgrobacterium-mediated transformation of Arabidopsis thaliana. Plant J..

[46] Cheng N.-H. (2008). AtGRX4, an Arabidopsis chloroplastic monothiol glutaredoxin, is able to suppress yeast grx5 mutant phenotypes and respond to oxidative stress. FEBS Lett..

[47] Karimi M., Inzé D., Depicker A. (2002). GATEWAY™ vectors for Agrobacterium-mediated plant transformation. Trends Plant Sci..

[48] Alonso J.M., Stepanova A.N., Leisse T.J., Kim C.J., Chen H., Shinn P., Stevenson D.K., Zimmerman J., Barajas P., Cheuk R. (2003). Genome-Wide Insertional Mutagenesis of Arabidopsis thaliana. Science.

[49] Johansen L.K., Carrington J.C. (2001). Silencing on the Spot. Induction and Suppression of RNA Silencing in the Agrobacterium-Mediated Transient Expression System. Plant Physiol..

[50] Jefferson R.A., Kavanagh T.A., Bevan M.W. (1987). GUS fusions: Beta-glucuronidase as a sensitive and versatile gene fusion marker in higher plants. EMBO J..

[51] Murashige T., Skoog F. (1962). A Revised Medium for Rapid Growth and Bio Assays with Tobacco Tissue Cultures. Physiol. Plant..

