# Supplementary material for: An Arabidopsis Oxalyl-CoA Decarboxylase, AtOXC, Is Important for Oxalate Catabolism in Plants
Source: Int J Mol Sci. 2021 Mar 23;22(6):3266. doi: 10.3390/ijms22063266 (PMC8004701; doi:10.3390/ijms22063266)
Supplement: Supplementary file 1 [file ijms-22-03266-s001.pdf]

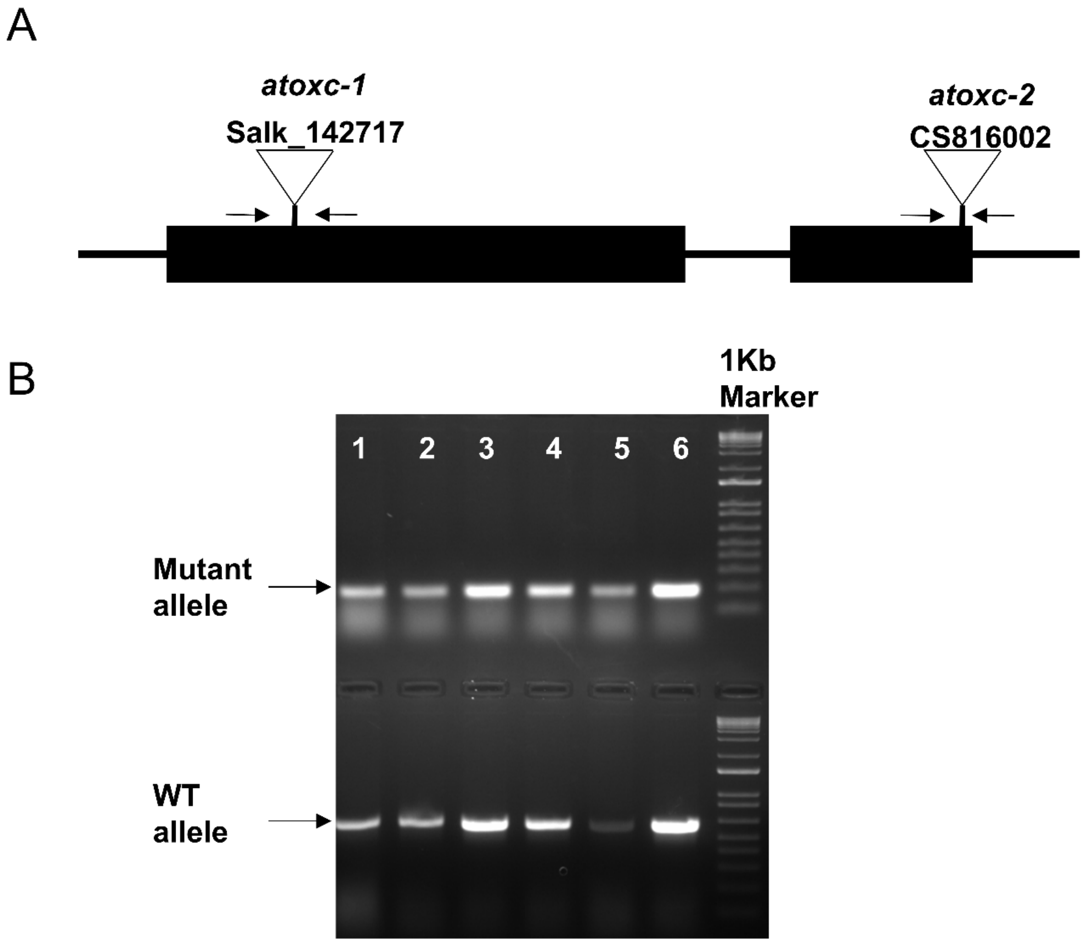

Figure S1. Analysis of two T-DNA insertional lines of *AtOXC* gene. (**A**) Diagram indicates the first allele (Salk_142717) and the second allele (SAIL_343_D06) of AtOXC T-DNA insertional lines. Arrows indicate the genotyping PCR primers. (**B**) PCR genotyping of Basta resistant individual plants of T3 SAIL_343_D06 line, showing all individual plants carry both T-DNA insertion and WT allele.
